# Electrocardiographic Imaging as Preoperative Tool in Persistent and Long-Standing Persistent Atrial Fibrillation: A Prospective Observational Study

**DOI:** 10.1093/icvts/ivaf198

**Published:** 2025-08-19

**Authors:** Emilio Osorio-Jaramillo, Luca Conci, Thomas Schlöglhofer, Iuliana Coti, Andreas Strassl, Christoph Schukro, Daniel Zimpfer, Marek P Ehrlich, Niv Ad

**Affiliations:** Department of Cardiac and Thoracic Aortic Surgery, Medical University of Vienna, A-1090 Vienna, Austria; Department of Cardiac and Thoracic Aortic Surgery, Medical University of Vienna, A-1090 Vienna, Austria; Department of Cardiac and Thoracic Aortic Surgery, Medical University of Vienna, A-1090 Vienna, Austria; Center for Medical Physics and Biomedical Engineering, Medical University of Vienna, A-1090 Vienna, Austria; Department of Cardiac and Thoracic Aortic Surgery, Medical University of Vienna, A-1090 Vienna, Austria; Division of Cardiovascular and Interventional Radiology, Medical University of Vienna, A-1090 Vienna, Austria; Department of Cardiac and Thoracic Aortic Surgery, Medical University of Vienna, A-1090 Vienna, Austria; Department of Cardiac and Thoracic Aortic Surgery, Medical University of Vienna, A-1090 Vienna, Austria; Department of Cardiac and Thoracic Aortic Surgery, Medical University of Vienna, A-1090 Vienna, Austria; Department of Cardiac and Thoracic Aortic Surgery, Medical University of Vienna, A-1090 Vienna, Austria; Division of Cardiac Surgery, Johns Hopkins University, 21218 Baltimore, MD, United States

**Keywords:** atrial fibrillation, electrocardiographic imaging, non-invasive mapping, surgical ablation

## Abstract

**Objectives:**

Surgical ablation for treating atrial fibrillation (AF) is currently performed mostly without preoperative electrophysiological imaging. This study aimed to investigate the use of non-invasive surface mapping as a preoperative tool to explore potential mechanisms and patterns involved in the electrophysiology of persistent and long-standing persistent AF.

**Methods:**

This prospective, observational study included cardiac surgery candidates without previous ablation. Bi-atrial epicardial activation sequences were obtained with electrocardiographic imaging and analysed in an independent core lab. Statistical analyses included hierarchical clustering, which quantified 3 clusters based on the number of drivers to identify specific characteristics.

**Results:**

All 51 patients [14 (27%) persistent; 37 (73%) long-standing persistent; AF, duration 42 months (interquartile range 14-120)] had bi-atrial electrophysiological abnormalities. Most rotors were harbored in the upper half of the right atrium, involved in almost all patients (50/51, 98%), followed by the pulmonary vein areas and left-atrial backwall (48/51, 94%). Longer AF duration showed no association towards fewer rotor and focal activity (*r* = −0.08, *P* = 0.42; *r* = −0.06, *P* = 0.56, respectively). A significant correlation existed between larger left atrial (LA) size and fewer rotors (*r* = −0.33, *P* < 0.001), but not focal activity (*r* = 0.01, *P* = 0.92). The clusters differed in AF duration and LA size, and in their number of rotor and focal activities (*P* = 0.005, *P* < 0.001, respectively).

**Conclusions:**

The underlying electrophysiological mechanism was identified in all patients and consistently showed bi-atrial involvement irrespective of AF duration, LA size, or concomitant heart disease. In larger left atria and longer AF duration, the observed lower number of rotors might be related to atrial tissue fibrosis and lower amplitudes. The results demonstrate the potential role of preoperative mapping to improve procedural planning and our understanding of patients’ electrophysiology.

**Clinical Registration:**

ClicalTrials.gov NCT06803615.

## Graphical abstract

**Figure:**
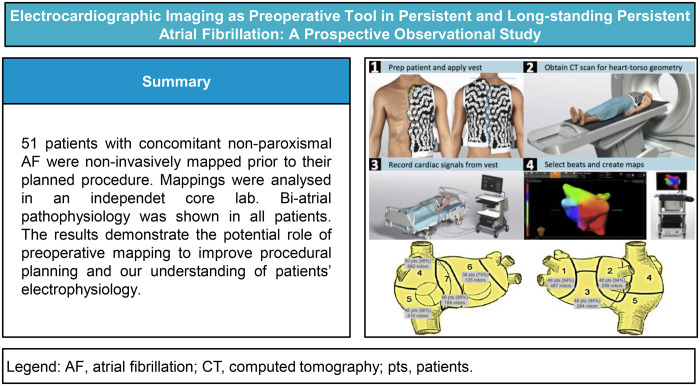

## INTRODUCTION

Decision-making related to the surgical approach and solutions during cardiac surgical procedures relies on accurate preoperative imaging. For example, prior to coronary artery bypass grafting (CABG), patients routinely undergo coronary angiography; the images serve to identify the coronary lesions and plan the procedure. Unlike any other cardiac surgical procedures, patients with atrial fibrillation (AF) that are candidates for concomitant surgical ablation (SA) present to surgery without specific information on the pathophysiology of the arrhythmia. This is a unique situation entailing that the planning of the ablation relies mostly on the nature of the concomitant procedure and type of AF, and to a lesser extent on the left atrial (LA) size and duration of AF. This constitutes a challenge that may hamper our ability to deliver the best long-term outcome in the safest way.

**Figure 1.**
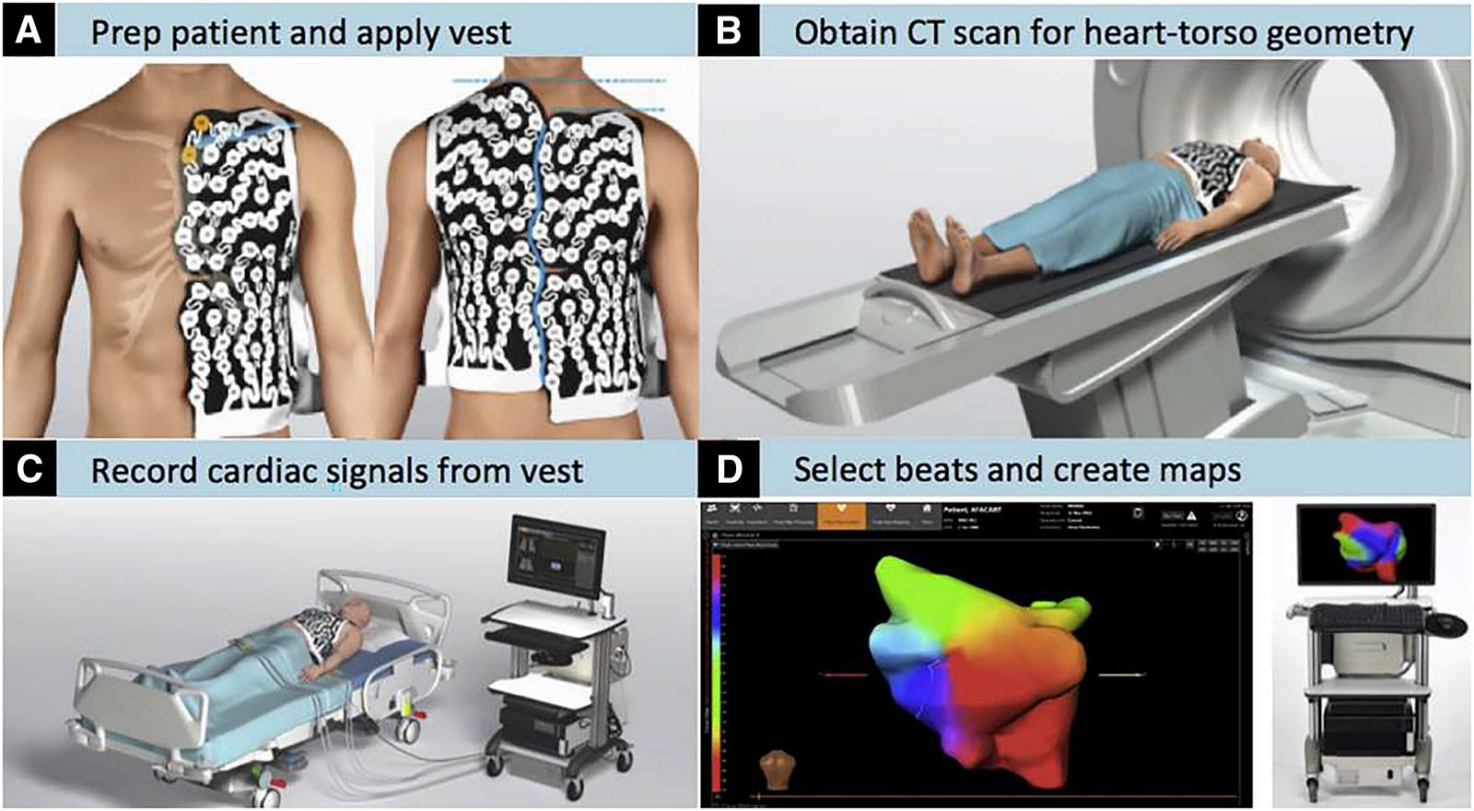
ECGI System. ECGI study: (A) The electrode vest is put onto shaved, dry skin, (B) CT scan, (C) recording, (D) 3D-reconstruction and reconstruction of the atrial surface potentials by the system’s algorithms. Figure reproduced with permission from the authors4 and Elsevier

Electrophysiologists can invasively perform intracardiac catheter-based electrophysiological mapping during the same ablation session to ascertain the pathophysiology of AF and plan their ablation strategy—an approach surgeons cannot pursue. Electrocardiographic imaging (ECGI), however, is a non-invasive mapping modality performed under close-to-physiological conditions, which allows for simultaneous recording of bi-atrial activation sequences,^1^ and can be easily performed prior to a planned procedure.^2^ ECGI can be done in an outpatient setting, patients are only required to wear a vest with an array of 252-electrodes for usually under 40 minutes, during signal acquisition and to undergo a non-contrast computed tomography (CT), all without sedation (**Figure 1**). Prior studies have demonstrated the feasibility of ECGI in mapping AF substrates^3^; however, data on its clinical utility in preoperative patients undergoing concomitant procedures for structural heart disease remain limited.^2^^,^^4–6^

The objective of this prospective, observational study was to assess the potential of ECGI as a preoperative mapping tool and to improve our understanding of patients’ electro-pathophysiology.

## METHODS

The study was conducted in accordance with the principles of the Declaration of Helsinki and the Declaration of Taipei. The Medical University of Vienna Ethics Review Board gave approval (No.EK1046/2018). All participants gave written informed consent prior to enrollment.

### Study design and population

This single-centre, open, prospective, observational study included eligible patients between January 2017 to January 2024 at Medical University of Vienna. Inclusion criteria: patients (>18 years) presenting with persistent (PeAF) or long-standing persistent AF (LSpAF)^7^ and no previous catheter or SA scheduled for cardiac surgery were invited to participate in the study. Following written and informed consent, patients underwent ECGI prior to their planned procedure. **Table 1** summarizes the patient characteristics. Forty-four (86%) patients had a planned surgical mitral valve procedure (**Table S4** lists concomitant disease specifics).

**Table 1.** Patient Characteristics and Clusters

Variables	All patients (*n *= 51)	Cluster 1 (*n *= 21)	Cluster 2 (*n *= 9)	Cluster 3 (*n *= 21)	*P*-values
Age, years, median (IQR)	71 (64-77)	70 (65-77)	67 (54-78)	73 (64-77)	0.73
Female sex	20 (39)	7 (33)	6 (67)	7 (33)	0.18
Atrial fibrillation duration, months, median (IQR)	42 (14-120)	60 (13-104)	24 (6-45)	48 (20-136)	0.26
Long-standing persistent AF	37 (73)	15 (71)	6 (67)	16 (76)	0.92
Persistent AF	14 (27)	6 (29)	3 (33)	5 (24)	
Antiarrhythmic drugs					
Beta blocker	38 (75)	15 (71)	7 (78)	16 (76)	0.99
Digitalis	6 (12)	1 (5)	2 (22)	3 (14)	0.38
Amiodarone	3 (6)	2 (10)	0 (0)	1 (5)	0.56
Vernakalant	2 (4)	2 (10)	0 (0)	0 (0)	0.22
None	7 (14)	4 (19)	0 (0)	3 (14)	0.36
Concomitant cardiac disease					
MR ≥°2	35 (69)	15 (71)	6 (67)	14 (67)	0.94
TR ≥°2	31 (61)	13 (62)	5 (56)	13 (62)	0.94
MS ≥°2	7 (14)	3 (14)	1 (11)	2 (10)	0.89
AS ≥°2	5 (10)	3 (14)	0 (0)	2 (10)	0.48
AR ≥°2	6 (12)	0 (0)	1 (11)	5 (24)	0.06
AAA	6 (12)	3 (14)	0 (0)	3 (14)	0.48
CAD	10 (20)	5 (24)	0 (0)	5 (24)	0.26
Ejection fraction					
>55%	38 (75)	17 (81)	5 (56)	12 (57)	0.13
45%-55%	12 (23)	2 (10)	3 (33)	7 (33)	
35%-45%	0 (0)	0 (0)	0 (0)	0 (0)	
<35%	1 (2)	0 (0)	1 (11)	0 (0)	
MR severity					
0 (none/trivial)	7 (14)	3 (14)	1 (11)	3 (14)	0.77
1 (mild)	9 (17)	3 (14)	2 (22)	4 (19)	
2 (moderate)	4 (8)	1 (5)	1 (11)	2 (10)	
3 (moderate-severe)	27 (53)	11 (52)	4 (45)	12 (57)	
4 (severe)	4 (8)	3 (14)	1 (11)	0 (0)	
Left atrial size, mm, median (IQR)	53 (44-59)	54 (47-62)	44 (39-51)	54 (45-60)	**0.026** 1 vs.2 (0.022^a^)
Right atrial size, mm, median (IQR)	60 (53-71)	65 (55-73)	63 (51-68)	56 (51-68)	0.29
Diabetes mellitus	7 (14)	3 (14)	1 (11)	3 (14)	0.97
COPD	4 (8)	6 (29)	1 (11)	1 (5)	0.097
Number of focal activities	3.9 (0.6-7.7)	2.6 (0-5.6)	2.5 (1.0-3.9)	6.9 (3.8-9.7)	**0.005** 1 vs.3 (0.01^a^) 2 vs.3 (0.05^a^)
Number of involved focal regions	2 (1-2)	1 (0-2)	2 (1-2)	2 (1-2.5)	0.07
Number of rotor activities	31.2 (23-46.9)	14.5 (11.2-25.3)	60.2 (54.1-70.5)	36.4 (30.8-43.9)	**<0.001** 1 vs. 3 (<0.001^a^) 1 vs. 2 (<0.001^a^) 2 vs. 3 (0.04^a^)
Number of involved rotor regions	7 (6-7)	6 (5-7)	7 (6.5-7)	7 (6-7)	**0.005** 1 vs. 3 (0.012^a^) 1 vs. 2 (0.031^a^)
Number of left sided rotor activities	22 (15-31)	15 (11-17)	45 (35-52)	25 (21-31)	**<0.001** 1 vs. 3 (<0.001^a^) 1 vs. 2 (<0.001^a^) 2 vs. 3 (0.0023^a^)
Number of right sided rotor activities	19 (11-32)	9 (5-15)	40 (34-48)	25 (18-32)	**<0.001** 1 vs. 3 (<0.001^a^) 1 vs. 2 (<0.001^a^) 2 vs. 3 (0.047^a^)
Atrio-ventricular valve related pathology	41 (80)	17 (81)	8 (89)	16 (76)	0.72

Values are *n* (%) unless indicated otherwise.

a Indicate Bonferroni correction. Bold values indicate statistically significant results.

Abbreviations: AAA: aneurysm of the ascending aorta; AF: atrial fibrillation; AR: aortic valve regurgitation; AS: aortic valve stenosis; CAD: coronary artery disease; COPD: chronic obstructive pulmonary disease; IQR: interquartile range; MR: mitral valve regurgitation; MS: mitral valve stenosis; TR: tricuspid valve regurgitation.

### Mapping technique

ECGI was carried out using the CardioInsight Non-invasive 3 D-Mapping System (Medtronic Inc., Minneapolis, MN, USA).^8^ To record body-surface potentials, a 252-electrode vest was applied to the patient’s torso (**Figure 1**). Signal acquisition was followed by a thoracic CT scan (third-generation dual-source CT, SOMATOM Force, Siemens Healthineers, Forchheim, Germany) to obtain high-resolution images of the heart and vest electrodes. Three-dimensional epicardial atrial geometries were manually reconstructed from segmental CT images. With the aid of well-validated mathematical algorithms, the system reconstructed epicardial potentials, unipolar electrograms, potential activation, and directional activation maps.^1^^,^^9^^,^^10^

In this and previous studies^2^^,^^3^^,^^11^^,^^12^ with this system, the term “driver”^13^ is used for the 2 potential types of electrical mechanisms that sustain, rather than initiate, fibrillatory conduction: focal activations and circulating wavefronts. Both can be visualized by phase analysis and categorized as either focal or rotor activity (example in **Figure S1**). All detections have spatial/temporal constraints applied in the system.

- Focal activity is defined as an activation arising from a single stable point radiating outwards.
- Rotor activity is defined as a wave rotating a minimum of 1.5 times around a spatially stable core.^3^

Stable rotors were identified and categorized as such only if they performed a rotational movement around a stable core.

All recordings were sent to a core laboratory in a blinded fashion for processing, which required 90 minutes in average. Every rotational activity was checked for plausibility during review and confirmed after sequential activation of the unipolar electrograms, covering the local cycle length around a pivot point. Focal activity detections were reviewed to remove potential detections due to far-field projections. A sample of 2 patients showing rotor activity and their respective electrograms are provided in **Videos 1 and 2**.

**Figure 2.**
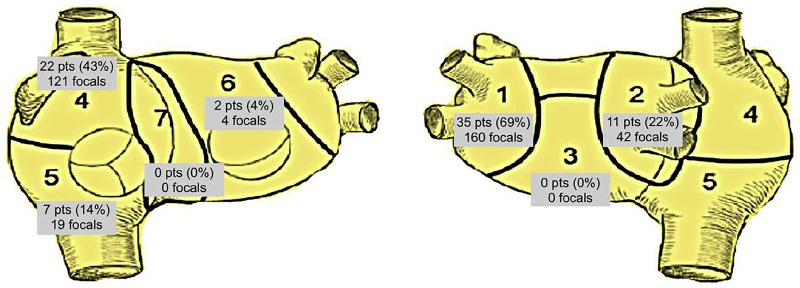
Overall Distribution of Focal Activities According to the Bordeaux Classification. The CT-based atrial geometry is divided into 7 regions. Areas 1, 2, 3, and 6 correspond to the left atrium. Areas 4 and 5 correspond to the right atrium and 7 covers the anterior interatrial groove

**Figure 3.**
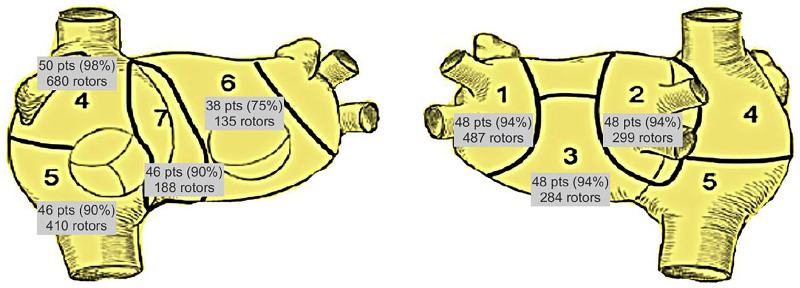
Overall Distribution of Rotor Activities According to the Bordeaux Classification

The Bordeaux atrial region classification^3^ was applied for bi-atrial regional subdivision into 7 areas (**Figures 2 and 3**).

### Cluster analysis

To compare quantitative results between subgroups and obtain homogeneity, the number of drivers was calculated for 10 recorded seconds for each patient.

A hierarchical cluster analysis determined the number of clusters for a k-means analysis to further identify patient characteristics based on their number of drivers. The resulting dendrogram (**Figure S3**) was used to establish the number of clusters for input into the k-means algorithm. To ascertain the optimal cluster assignments for patients, an iterative approach to k-means clustering was employed to minimize the Euclidean distance between data points and their corresponding cluster centroids.^14^ Eight iterations were used to minimize variances within clusters. After each iteration, the algorithm dynamically adjusted the centroid values, continuing until they converged and remained constant.^15^

### Statistical analysis

SPSS 29.0.0 (IBM, NY, USA) was used for statistical analyses. Absolute numbers (percentages) were reported for categorical variables, and medians (interquartile ranges [IQRs]) for continuous variables. Continuous variables underwent normal distribution testing using the Shapiro-Wilk-test. Kendall’s tau correlation was utilized to explore the relationship between individuals’ AF duration or atrial size and the number of drivers. Baseline characteristics, risk factors, and mapping results were compared among clusters using the Fisher’s exact test for categorical variables and the Kruskal-Wallis test for independent continuous variables, followed by a Bonferroni correction for multiple comparisons. Two-sided *P*-values <0.05 were defined as statistically significant.

## RESULTS

Fifty-one consecutive patients with PeAF and LSpAF were included in this study. Mapping was performed a minimum of 1 day before their intervention (median 7 days, IQR 2-17). Median AF duration was 42 months (IQR 14-120).

Overall, a total of 765 phase windows (median 14 per patient [pp], IQR 13-16) were available for analysis, which translated into 766 534 ms of recording time (median 14 775 ms pp, IQR 13 990-15 977).

In total, 346 focal activities and 2483 rotors were recorded. **Tables S1 and S2** show the exact distribution of driver activity per patient and area. The median driver activity per area is shown in **Table S3**.

### Focal activity

Focal activity was detected in 42 patients (82%). The median number of focals per patient was 6 (IQR 1-11). Twenty-two patients showed biatrial involvement (43%).

The majority of focal activities were detected in area 1 [the left upper and lower pulmonary veins (PVs)] showing a total of 160 focals in 35 patients (69%), followed by area 4 [right atrial (RA) appendage and superior vena cava (VCS) region] with 121 focals in 22 patients (43%). The right upper and lower PVs showed 42 focals in 11 patients (22%). It is worth mentioning that we did not find a single focal activation in areas 3 (posterior wall of LA) and 7 (anterior interatrial groove). The distribution of the overall mapped focal activities according to their area is depicted in **Figure 2**.

### Rotor activity

Rotor activity was consistently documented in every mapped individual (51, 100%), and during every study. Moreover, rotors were always present in both atria in all patients. Thus, there was no instance of rotors in only 1 atrium (detailed distribution in **Figure 3**). The median number of rotors was 46 (IQR 27-61).

Interestingly, the area involving rotor activity in almost all patients was the upper half of the RA (area 4) with 50 patients (98%). The upper half of the RA was also the area with the highest cumulative (680 rotors) and median numbers (13pp, IQR 6-19) of rotors. The subsequent most common regions for the detection of rotor activity as defined by number of patients and total number of rotors were area 1 (left upper and lower PVs and LA appendage) with a cumulative number of 487 rotors, and area 2 (right-sided PV and posterior interatrial groove) with 299 rotors, in 48 patients (94%) each. The LA backwall (area 3) accounted for 284 rotors in a third group of 48 patients (94%). The area with the third highest number of rotors (410) was the lower half of the RA (area 5) involving 46 patients (90%).

### AF duration and atrial size

We sought to determine whether the patients’ AF duration or atrial size correlated with the number of drivers.

There was no association between rotor and focal activities in patients with longer AF duration (**Figure S2**; *r* = −0.08, *P* = 0.42; *r* = −0.06, *P* = 0.56, respectively). Longer AF duration did not correlate with a higher number of affected atrial regions (focals: *r* = −0.1, *P* = 0.35; rotors: *r *= 0.03, *P* = 0.80).

**Figure 4.**
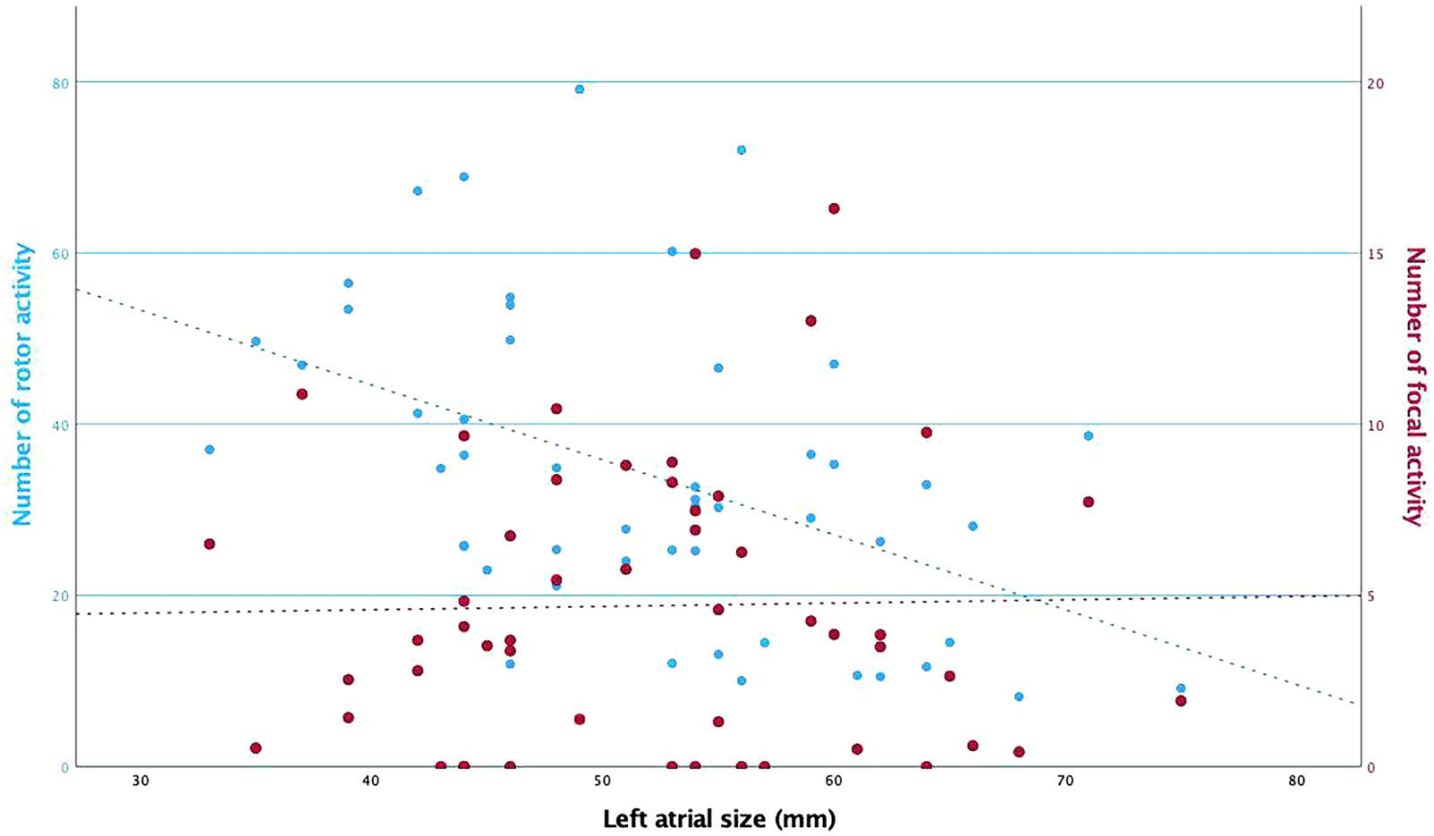
Relationship Between Atrial Size and Number of Rotor and Focal Activities

There was a statistically significant correlation between larger LA size and a lower number of rotors (*r* = −0.33, *P* < 0.001), but no correlation was found between LA size and the number of focals (*r* = 0.01, *P* = 0.92), see **Figure 4**. Furthermore, no correlation between LA size and the number of involved atrial regions (focals: *r* = −0.002, *P* = 0.99; rotors: *r* = −0.05, *P* = 0.68) was found.

### Cluster analysis

To further identify each patient’s number of mapped drivers, a means cluster analysis was performed. The resulting dendrogram (**Figure S3**) was divided into 3 clusters.

The cluster’s characteristics are summarized in **Table 1**. *Clusters 1* and *3* each comprised 21 patients (41%, respectively), while *cluster 2* comprised 9 patients (18%). The 3 clusters differed in their total number of involved rotor and focal activities (*P* = 0.005, *P* < 0.001, respectively).

**Figure 5.**
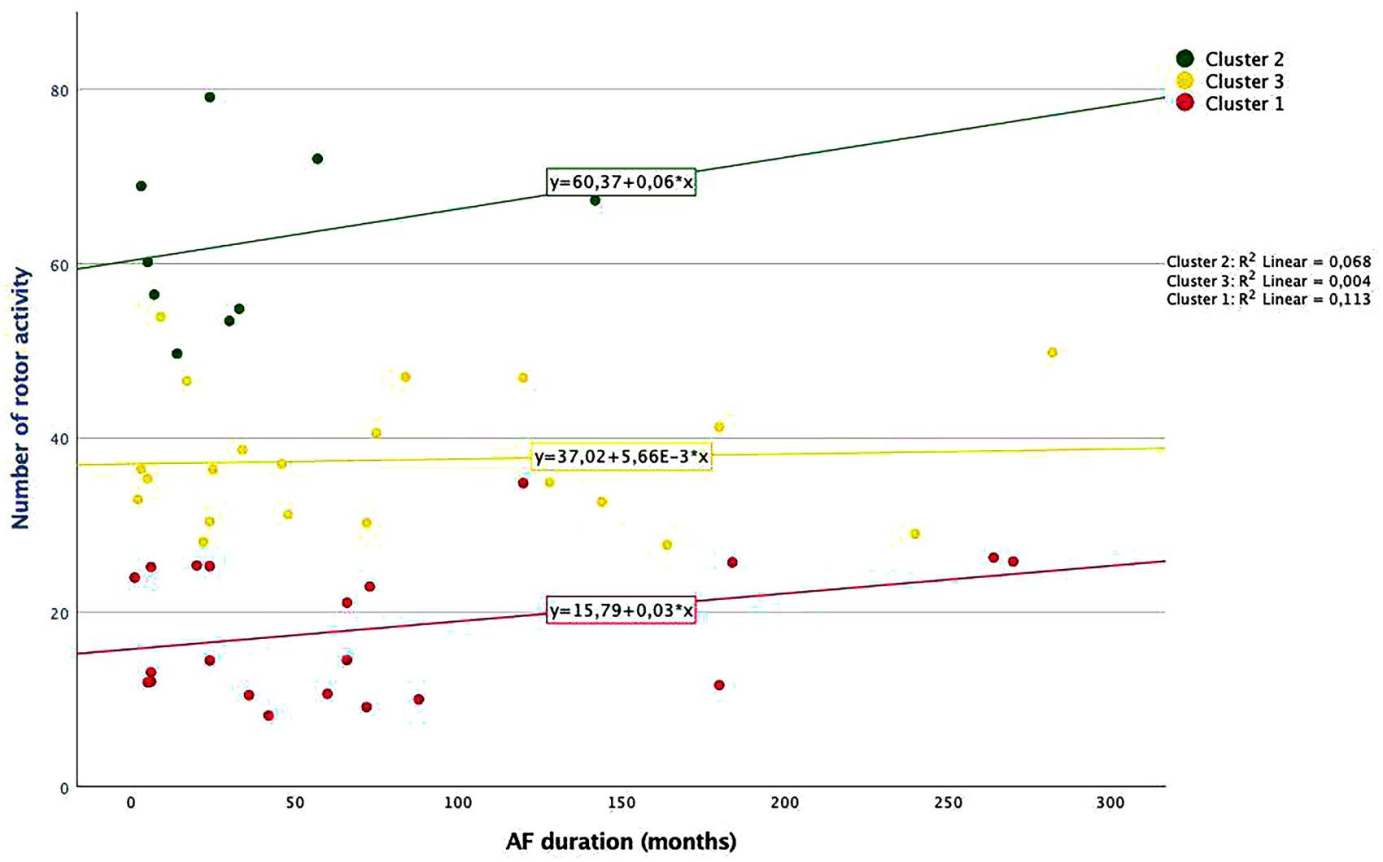
Relationship Between AF Duration and Number of Rotor Activities for Each Cluster. Abbreviation: AF: atrial fibrillation

For every cluster, the relationship between the number of rotors and their AF duration is depicted in **Figure 5**.

Patients in *cluster 1* showed the lowest number of rotors (<0.001) and fewer involved rotor regions. This group of patients numerically had the longest median AF duration (60 months, IQR 13-104, *P* = 0.26), with larger LA than patients in *cluster 2* (*P* = 0.022).

In *cluster 2,* patients had the smallest median LA size (44 mm, IQR 39-51) and numerically the shortest AF duration (24 months [IQR 6-45]), as well as the highest number of re-entrant drivers (*P* < 0.001) (see **Figure 5**). This was the case for overall numbers and for the LA and RA.

Patients in *cluster 3* were between cluster 1 and 2 concerning AF duration and rotor numbers. This group had a similar median LA size as *cluster 1* but showed significantly more focal activities.

## DISCUSSION

This study produced very interesting results that, if found consistent in a larger group of patients, should impact our approach to concomitant SA for AF. We summarized for the first time the electrophysiological mechanism of a large collective of patients with non-paroxysmal AF. All patients were mapped with no technical issues and ECGI produced excellent quality maps. Bi-atrial electrical activity was captured in all patients together with significant AF-atrial substrates beyond just the PVs and the left atrium. An abundance of rotors involved patients’ whole posterior LA walls, PV antral regions, as well as the upper part of the RA and its appendage. Those findings were independent of AF duration and underlying cardiac pathology.

Additionally, a hierarchical cluster analysis determined the number of clusters and appropriate starting seeds (the initial distribution points), followed by a k-means analysis to refine the results.^16^ The k-means analysis, a type of unsupervised machine-learning, enabled labelling of input data and uncovered relationships and trends in our data that would not have been readily apparent in simple visual examination.

This helped to make 2 important observations. It showed that patients with larger LA were more likely to have fewer rotors, and it showed that patients with the longest AF duration had fewer driver activities possibly related to atrial tissue fibrosis and lower amplitudes, as LSpAF has been associated with a significant degree of atrial tissue remodelling.^7^ This may explain the lower success rates of certain SA approaches observed in patients with larger atria and longer AF duration.

This is the largest study to-date reporting preoperatively mapped patients with PeAF and LSpAF. We chose to use ECGI, because preoperative catheter-mapping would require an additional procedure under anaesthesia which might be too burdensome for most patients. Contrary to traditional invasive EP (electrophysiological)-studies, ECGI is a non-invasive tool and the only currently available mapping technique allowing for simultaneous recording of both atria under near-normal physiological conditions. A vest with a 252-electrode array is worn during recording of the arrhythmia and CT scanning to obtain heart and torso geometries for subsequent 3 D-reconstruction. This technique is safe and easy to apply before cardiac procedures because throughout this process patients are awake and need no sedation. This prevents potential sedation-related alteration of mapping results and/or haemodynamic compromise of preoperative, vulnerable patients.

Extensive research has led to the development of this technology,^1^ now used to aid interventional ablation procedures.^3^^,^^11^^,^^12^ ECGI has also been applied in various experimental settings including single-beat mapping of sinus rhythm to assess atrial conduction^6^^,^^17^ and has been shown to be accurate when compared with intracardiac catheter mapping.^18^^,^^19^ Only a limited number of studies has used this technology for the mapping of preoperative patients with AF.^2^^,^^4^^,^^5^ Santer et al^5^ showed, in a retrospective analysis of their cohort of 13 preoperative patients with non-paroxysmal AF (all mapped patients showed rotor activity), that performing ECGI led to an increased used of SA as compared to a control group of patients that did not receive preoperative mapping. Additionally, the use of ECGI led to a higher rate of biatrial ablation if SA was performed.^5^

We hypothesized that ECGI would be a suitable method to help identify patients’ electrophysiological mechanisms involved in their arrhythmia prior to surgery.

Concomitant SA is currently performed mostly without preoperative mapping. The procedure is therefore performed without knowledge of the underlying electrophysiological mechanism. Conversely, in aortic surgery, patients usually require a CT or magnetic resonance imaging (MRI)-scan, and in coronary surgery, every CABG is usually planned following coronary angiography. Preoperative imaging helps surgeons plan the appropriate procedure, which is key for a successful outcome. In SA, this type of approach is not yet established.

Some groups have used intraoperative epicardial mapping in the setting of a study, such as those conducted by Nitta et al^20^ and Sahadevan et al^21^ and have provided significant insights into AF mechanisms. For example, Sahadevan et al identified regions of rapid, regular activation, consistent with potential arrhythmogenic drivers that sustain fibrillatory conduction. Similarly, Nitta et al demonstrated the utility of intraoperative mapping to guide SA strategies tailored to patient-specific electrophysiological patterns, achieving high success rates. While intraoperative mapping offers unparalleled spatial resolution and the ability to integrate directly with surgical interventions, ECGI expands the scope of arrhythmia evaluation to preoperative and by enabling preoperative planning.

In SA, surgeons often work alone and not as part of a heart-team; so they tend to treat the LA only or perform a simple PV isolation (PVI), without EP support or follow-up planning.^22^^,^^23^ Our study should be seen as an incentive to explore potential new avenues in the area of SA, as for example through new collaborative hybrid concepts in which a treatment plan is being generated after taking into account all clinical and electrophysiological parameters during a heart-team meeting. Preoperative mapping could help pursue such different treatment strategies and facilitate surgical decision-making through preoperative planning when surgeons either consider it too risky to perform a complete Cox-maze procedure or, consensus exists that a hybrid procedure would be advantageous for the specific patient, for example, when a minimally invasive access or a shorter operation and improved long-term AF-ablation success is pursued. Preoperative mapping could facilitate such decisions and procedural planning by offering improved understanding of a patient’s specific electrophysiological mechanism.

In non-paroxysmal AF, defining the mechanisms and the extent of atrial remodelling involved is a matter of ongoing debate.^24^ The identified mechanisms include multiple wavefronts and localized drivers. The reported AF-mechanisms, their initiation, and propagation depended on the patient sub-sets and mapping techniques applied, these being common techniques using activation and voltage mapping, for example, through endo- and epicardial mapping.^25–32^ The mechanisms involved in the initiation of paroxysmal AF are simpler, especially in patients with a shorter history of AF,^33^ and treatment via PVI has proven to be safe and sufficiently effective for most patients.^7^^,^^34^ This is not the case for PeAF and LSpAF.

Depending on the underlying concomitant pathology, AF prevalence and the likelihood to undergo SA varies.^35^^,^^36^ STS-registry data showed that patients requiring mitral valve surgery had the highest likelihood of receiving some type of SA (61.5%), compared to aortic valve replacement (AVR) (33.9%) and CABG (27.5%); however, those numbers are even lower, based on results of the Medicare database,^23^ the Polish registry,^37^ and data generated in the State of Maryland.^38^ The hurdle to address AF seems to be higher in procedures like AVR or CABG vs procedures where the atria are opened anyway.^23^ This is reflected in the observed higher rate of SA performed during mitral valve operations.^36^ Our collective included patients with mitral and tricuspid valve pathologies, but also a smaller group of patients with non-atrioventricular-valve related pathologies. We believe that this latter collective of patients would specifically benefit from preoperative electrophysiological assessment to plan an optimal SA procedure and long-term post-procedural approaches.

However, a larger multicentre study and more patients would be needed to provide specific treatment recommendations and to fully cover the spectrum of the surgical patient population.

Finally, the results of our study suggest that preoperative “imaging” of a patient’s AF mechanism provides essential information so that preoperative assessment of patients with AF should include some sort of electrophysiological study. Furthermore, we strongly believe that collaboration and planning with an electrophysiologist are of utmost importance for delivering AF treatment to more patients, facilitating and potentially even accelerating the surgical procedure and improving outcomes.

### Limitations

This study was performed in a heterogeneous group of patients, with a high proportion of LSpAF. There are some limitations to ECGI, which determines the heart’s electrical sources for a given body-surface potential distribution. This is also known as the inverse problem, which is considered ill-posed.^39^ ECGI was successfully performed in all patients without technical difficulties, yielding excellent-quality maps. We believe that possible disadvantages of this technique, such as the somewhat lower subsurface resolution compared to the resolution gained with invasive mapping, are offset by the possibility to easily map most preoperative patients. However, the accuracy of ECGI has been shown to be of 6 mm.^18^

## CONCLUSION

This study tested the concept of preoperative mapping in patients with AF and is the largest study documenting rotor and focal activation patterns in preoperative candidates with non-paroxysmal AF utilizing ECGI. Bi-atrial pathophysiology was shown in all patients, irrespective of AF duration, LA size, or underlying heart disease. In patients with longer AF duration and larger atria, a lower number of drivers might be related to atrial tissue fibrosis and lower amplitudes. These results underscore the importance of understanding the potential AF mechanism preoperatively in order to better address it surgically. This would enable applying hybrid concepts determined by the heart-team, involving an electrophysiologist, for optimized treatment of concomitant AF.

## Supplementary Material

ivaf198_Supplementary_Data

## Data Availability

The data underlying this article will be shared on reasonable request to the corresponding author.

## References

[1] Rudy Y. Noninvasive electrocardiographic imaging of arrhythmogenic substrates in humans. Circ Res. 2013;112:863-874. 23449548 10.1161/CIRCRESAHA.112.279315 PMC3596167

[2] Osorio-Jaramillo E, Cox JL, Klenk S, et al Dynamic electrophysiological mechanism in patients with long-standing persistent atrial fibrillation. Front Cardiovasc Med. 2022;9:953622. 36247427 10.3389/fcvm.2022.953622 PMC9556291

[3] Haissaguerre M, Hocini M, Denis A, et al Driver domains in persistent atrial fibrillation. Circulation. 2014;130:530-538. 25028391 10.1161/CIRCULATIONAHA.113.005421

[4] Ehrlich MP, Laufer G, Coti I, et al Noninvasive mapping before surgical ablation for persistent, long-standing atrial fibrillation. J Thorac Cardiovasc Surg. 2019;157:248-256. 30482525 10.1016/j.jtcvs.2018.07.104

[5] Santer D, Gahl B, Dogan A, et al Preoperative non-invasive mapping for targeted concomitant surgical ablation of non-paroxysmal atrial fibrillation (PreMap study). J Clin Med. 2025;14:481. 10.3390/jcm14020481 PMC11766366 39860487

[6] Schill MR, Vijayakumar R, Yates T-A, et al Sinus rhythm atrial electrocardiographic imaging in patients with mitral regurgitation: clues to the substrate for atrial fibrillation. Circ Arrhythm Electrophysiol. 2024;17:e012666. 38629291 10.1161/CIRCEP.123.012666 PMC11108731

[7] Joglar JA, Chung MK, Armbruster AL, et al 2023 ACC/AHA/ACCP/HRS guideline for the diagnosis and management of atrial fibrillation: a report of the American College of Cardiology/American Heart Association joint committee on clinical practice guidelines. Circulation. 2024;149:e1-e156. 38033089 10.1161/CIR.0000000000001193 PMC11095842

[8] Oster HS, Taccardi B, Lux RL, Ershler PR, Rudy Y. Noninvasive electrocardiographic imaging: reconstruction of epicardial potentials, electrograms, and isochrones and localization of single and multiple electrocardiac events. Circulation. 1997;96:1012-1024. 9264513 10.1161/01.cir.96.3.1012

[9] Ramanathan C, Ghanem RN, Jia P, Ryu K, Rudy Y. Noninvasive electrocardiographic imaging for cardiac electrophysiology and arrhythmia. Nat Med. 2004;10:422-428. 15034569 10.1038/nm1011 PMC1950745

[10] Rudy Y, Burnes JE. Noninvasive electrocardiographic imaging. Noninvasive Electrocardiol. 1999;4:340-359.

[11] Honarbakhsh S, Dhillon G, Abbass H, et al Noninvasive electrocardiographic imaging-guided targeting of drivers of persistent atrial fibrillation: the TARGET-AF1 trial. Heart Rhythm. 2022;19:875-884. 35134548 10.1016/j.hrthm.2022.01.042

[12] Knecht S, Sohal M, Deisenhofer I, et al Multicentre evaluation of non-invasive biatrial mapping for persistent atrial fibrillation ablation: the AFACART study. Europace. 2017;19:1302-1309. 28204452 10.1093/europace/euw168

[13] Baykaner T, Rogers AJ, Meckler GL, et al Clinical implications of ablation of drivers for atrial fibrillation: a systematic review and meta-analysis. Circ Arrhythm Electrophysiol, 2018;11:e006119. 29743170 10.1161/CIRCEP.117.006119 PMC6474343

[14] Han J, Pei J, Tong H. Data Mining: Concepts and Techniques. Morgan Kaufmann; 2022.

[15] Singh A, Yadav A, Rana A. K-means with three different distance metrics. IJCA. 2013;67:13-17.

[16] Clatworthy J, Buick D, Hankins M, Weinman J, Horne R. The use and reporting of cluster analysis in health psychology: a review. Br J Health Psychol. 2005;10:329-358. 16238852 10.1348/135910705X25697

[17] Eichenlaub M, Mueller-Edenborn B, Lehrmann H, et al Non-invasive body surface electrocardiographic imaging for diagnosis of atrial cardiomyopathy. Europace. 2021;23:2010-2019. 34463710 10.1093/europace/euab140

[18] Cuculich PS, Wang Y, Lindsay BD, et al Noninvasive characterization of epicardial activation in humans with diverse atrial fibrillation patterns. Circulation. 2010;122:1364-1372. 20855661 10.1161/CIRCULATIONAHA.110.945709 PMC2996091

[19] Rodrigo M, Climent AM, Hernández-Romero I, et al Noninvasive assessment of complexity of atrial fibrillation: correlation with contact mapping and impact of ablation. Circ Arrhythm Electrophysiol. 2020;13:e007700. 32078374 10.1161/CIRCEP.119.007700 PMC7508259

[20] Nitta T, Ohmori H, Sakamoto S-I, Miyagi Y, Kanno S, Shimizu K. Map-guided surgery for atrial fibrillation. J Thorac Cardiovasc Surg. 2005;129:291-299. 15678038 10.1016/j.jtcvs.2004.09.012

[21] Sahadevan J, Ryu K, Peltz L, et al Epicardial mapping of chronic atrial fibrillation in patients: preliminary observations. Circulation. 2004;110:3293-3299. 15520305 10.1161/01.CIR.0000147781.02738.13

[22] McCarthy PM, Cox JL, Kislitsina ON, et al Surgery and catheter ablation for atrial fibrillation: history, current practice, and future directions. J Clin Med. 2021;11:210. 35011953 10.3390/jcm11010210 PMC8745682

[23] McCarthy PM, Davidson CJ, Kruse J, et al Prevalence of atrial fibrillation before cardiac surgery and factors associated with concomitant ablation. J Thorac Cardiovasc Surg. 2020;159:2245-2253.e15. 31444073 10.1016/j.jtcvs.2019.06.062

[24] Schotten U, Lee S, Zeemering S, Waldo AL. Paradigm shifts in electrophysiological mechanisms of atrial fibrillation. Europace. 2021;23:ii9-ii13. 33837750 10.1093/europace/euaa384 PMC8035704

[25] Chen YL, Ban JE, Park YM, Choi JI, Park SW, Kim YH. The spatial distribution of atrial fibrillation termination sites in the right atrium during complex fractionated atrial electrograms-guided ablation in patients with persistent atrial fibrillation. J Cardiovasc Electrophysiol. 2013;24:949-957. 23773419 10.1111/jce.12187

[26] Child N, Clayton RH, Roney CH, et al Unraveling the underlying arrhythmia mechanism in persistent atrial fibrillation: results from the STARLIGHT study. Circ Arrhythm Electrophysiol. 2018;11:e005897. 29858382 10.1161/CIRCEP.117.005897

[27] Cox JL, Canavan TE, Schuessler RB II., et al The surgical treatment of atrial fibrillation. J Thorac Cardiovasc Surg. 1991;101:406-426. 1999934

[28] Lee S, Sahadevan J, Khrestian CM, Cakulev I, Markowitz A, Waldo AL. Simultaneous biatrial high-density (510–512 electrodes) epicardial mapping of persistent and long-standing persistent atrial fibrillation in patients. Circulation. 2015;132:2108-2117. 26499963 10.1161/CIRCULATIONAHA.115.017007 PMC4666790

[29] Nitta T, Ishii Y, Miyagi Y, Ohmori H, Sakamoto S, Tanaka S. Concurrent multiple left atrial focal activations with fibrillatory conduction and right atrial focal or reentrant activation as the mechanism in atrial fibrillation. J Thorac Cardiovasc Surg. 2004;127:770-778. 15001906 10.1016/j.jtcvs.2003.05.001

[30] Rostock T, Steven D, Hoffmann B, et al Chronic atrial fibrillation is a biatrial arrhythmia: data from catheter ablation of chronic atrial fibrillation aiming arrhythmia termination using a sequential ablation approach. Circ Arrhythm Electrophysiol. 2008;1:344-353. 19808429 10.1161/CIRCEP.108.772392

[31] Schmitt C, Ndrepepa G, Weber S, et al Biatrial multisite mapping of atrial premature complexes triggering onset of atrial fibrillation. Am J Cardiol. 2002;89:1381-1387. 12062732 10.1016/s0002-9149(02)02350-0

[32] Weipert KF, Hutter J, Kuniss M, et al Pulmonary vein isolation followed by biatrial ablation of rotational activity in patients with persistent atrial fibrillation: results of the cryo-vest study. J Clin Med. 2024;13:1118. 10.3390/jcm13041118 PMC10889131 38398432

[33] Haissaguerre M, Jais P, Shah DC, et al Spontaneous initiation of atrial fibrillation by ectopic beats originating in the pulmonary veins. N Engl J Med. 1998;339:659-666. 9725923 10.1056/NEJM199809033391003

[34] Ad N, Damiano RJ Jr., Badhwar V, et al Expert consensus guidelines: examining surgical ablation for atrial fibrillation. J Thorac Cardiovasc Surg. 2017;153:1330-1354.e1. 28390766 10.1016/j.jtcvs.2017.02.027

[35] Ad N, Suri RM, Gammie JS, Sheng S, O'Brien SM, Henry L. Surgical ablation of atrial fibrillation trends and outcomes in North America. J Thorac Cardiovasc Surg. 2012;144:1051-1060. 22920597 10.1016/j.jtcvs.2012.07.065

[36] Gammie JS, Haddad M, Milford-Beland S, et al Atrial fibrillation correction surgery: lessons from the Society of Thoracic Surgeons National Cardiac Database. Ann Thorac Surg. 2008;85:909-914. 18291169 10.1016/j.athoracsur.2007.10.097

[37] Kowalewski M, Jasiński M, Staromłyński J, et al Long-term survival following surgical ablation for atrial fibrillation concomitant to isolated and combined coronary artery bypass surgery-analysis from the Polish National Registry of Cardiac Surgery Procedures (KROK). J Clin Med. 2020;9:1345. 10.3390/jcm9051345 PMC7290935 32375414

[38] Ad N, Kang JK, Chinedozi ID, et al; Maryland Cardiac Surgery Quality Initiative Collaborative. Statewide data on surgical ablation for atrial fibrillation: the data provide a path forward. J Thorac Cardiovasc Surg. 2024;167:1766-1775. 37160217 10.1016/j.jtcvs.2023.04.020

[39] Salinet J, Molero R, Schlindwein FS, et al Electrocardiographic imaging for atrial fibrillation: a perspective from computer models and animal experiments to clinical value. Front Physiol. 2021;12:653013. 33995122 10.3389/fphys.2021.653013 PMC8120164

