# Supplementary material for: Electrocardiographic Imaging as Preoperative Tool in Persistent and Long-Standing Persistent Atrial Fibrillation: A Prospective Observational Study
Source: Interdiscip Cardiovasc Thorac Surg. 2025 Aug 19;40(9):ivaf198. doi: 10.1093/icvts/ivaf198 (PMC12548033; doi:10.1093/icvts/ivaf198)
Supplement: ivaf198_Supplementary_Data [file ivaf198_Supplementary_Data.zip › Supplementary Material.docx]

**Supplementary Material**

**Supplementary Table 1:** Number and distribution of focal activities. 2

**Supplementary Table 2:** Number and distribution of rotor activities. 4

**Supplementary Table 3:** Median overall driver activity. 6

**Supplementary Table 4:** Concomitant disease specifics. 7

**Supplementary Figure 1:** 8

Mapping study of patient no. 21 showing the distribution of drivers in LAO and PA view.

**Supplementary Figure 2:** 9

Relationship between AF duration and the number of divers.

**Supplementary Figure 3:** 10

Dendrogram of the hierarchical cluster analysis.

| **Supplementary Table 1: Number and distribution of focal activities.** | | | | | | | | |
| --- | --- | --- | --- | --- | --- | --- | --- | --- |
| **Patient no.** | **A1** | **A2** | **A3** | **A4** | **A5** | **A6** | **A7** | **∑** |
| 1 | 2 |  |  |  | 4 |  |  | 6 |
| 2 | 1 |  |  |  |  |  |  | 1 |
| 3 |  |  |  |  |  |  |  | 0 |
| 4 |  |  |  |  |  |  |  | 0 |
| 5 | 1 |  |  |  |  |  |  | 1 |
| 6 | 3 |  |  |  | 1 |  |  | 4 |
| 7 |  |  |  |  |  |  |  | 0 |
| 8 | 1 |  |  |  |  |  |  | 1 |
| 9 |  | 2 |  |  |  |  |  | 2 |
| 10 |  |  |  |  |  |  |  | 0 |
| 11 | 2 |  |  | 4 |  |  |  | 6 |
| 12 | 2 |  |  | 2 |  |  |  | 4 |
| 13 | 4 | 2 |  |  | 4 |  |  | 10 |
| 14 |  |  |  |  |  |  |  | 0 |
| 15 | 18 | 2 |  | 2 | 2 |  |  | 24 |
| 16 |  |  |  | 10 |  |  |  | 10 |
| 17 |  |  |  |  |  |  |  | 0 |
| 18 | 2 | 2 |  | 4 |  |  |  | 8 |
| 19 |  |  |  | 4 |  |  |  | 4 |
| 20 | 7 | 9 |  |  |  |  |  | 16 |
| 21 |  | 1 |  |  |  |  |  | 1 |
| 22 | 6 |  |  | 4 |  |  |  | 10 |
| 23 |  |  |  |  |  |  |  | 0 |
| 24 |  |  |  |  | 2 | 2 |  | 4 |
| 25 | 4 | 2 |  |  |  |  |  | 6 |
| 26 | 4 |  |  | 2 |  |  |  | 6 |
| 27 | 2 |  |  | 14 |  |  |  | 16 |
| 28 | 2 |  |  |  |  |  |  | 2 |
| 29 |  |  |  | 6 |  |  |  | 6 |
| 30 | 8 |  |  | 4 |  |  |  | 12 |
| 31 | 11 |  |  | 2 |  |  |  | 13 |
| 32 | 3 |  |  | 11 |  |  |  | 14 |
| 33 | 4 |  |  | 7 |  |  |  | 11 |
| 34 | 20 |  |  |  |  |  |  | 20 |
| 35 | 2 |  |  |  |  | 2 |  | 4 |
| 36 | 1 | 9 |  |  | 2 |  |  | 12 |
| 37 |  |  |  | 13 |  |  |  | 13 |
| 38 | 8 | 5 |  | 2 |  |  |  | 15 |
| 39 |  |  |  |  |  |  |  | 0 |
| 40 | 2 |  |  |  |  |  |  | 2 |
| 41 | 2 |  |  | 18 | 4 |  |  | 24 |
| 42 | 6 | 4 |  | 2 |  |  |  | 12 |
| 43 |  |  |  |  |  |  |  | 0 |
| 44 | 3 |  |  | 2 |  |  |  | 5 |
| 45 | 2 |  |  | 4 |  |  |  | 6 |
| 46 | 4 | 4 |  |  |  |  |  | 8 |
| 47 | 6 |  |  |  |  |  |  | 6 |
| 48 | 4 |  |  |  |  |  |  | 4 |
| 49 | 4 |  |  | 2 |  |  |  | 6 |
| 50 | 7 |  |  |  |  |  |  | 7 |
| 51 | 2 |  |  | 2 |  |  |  | 4 |
| **∑** | 160 | 42 | 0 | 121 | 19 | 4 | 0 | **346** |

Values are *n.* A: Area according to the Bordeaux atrial region classification. ∑: Sum.

| **Supplementary Table 2: Number and distribution of rotor activities.** | | | | | | | | |
| --- | --- | --- | --- | --- | --- | --- | --- | --- |
| **Patient no.** | **A1** | **A2** | **A3** | **A4** | **A5** | **A6** | **A7** | **∑** |
| 1 | 4 | 9 | 4 | 1 | 0 | 0 | 0 | 18 |
| 2 | 4 | 6 | 3 | 2 | 3 | 0 | 1 | 19 |
| 3 | 6 | 4 | 3 | 7 | 1 | 1 | 5 | 27 |
| 4 | 7 | 8 | 1 | 4 | 0 | 0 | 0 | 20 |
| 5 | 3 | 5 | 6 | 0 | 1 | 0 | 6 | 21 |
| 6 | 2 | 3 | 3 | 2 | 5 | 0 | 4 | 19 |
| 7 | 0 | 5 | 4 | 5 | 4 | 0 | 4 | 22 |
| 8 | 11 | 24 | 15 | 25 | 11 | 1 | 5 | 92 |
| 9 | 3 | 8 | 6 | 1 | 0 | 1 | 1 | 20 |
| 10 | 11 | 7 | 5 | 4 | 0 | 0 | 4 | 31 |
| 11 | 1 | 2 | 5 | 13 | 8 | 8 | 4 | 41 |
| 12 | 1 | 3 | 4 | 6 | 5 | 2 | 1 | 22 |
| 13 | 2 | 2 | 6 | 29 | 12 | 0 | 6 | 57 |
| 14 | 27 | 6 | 0 | 5 | 13 | 0 | 4 | 55 |
| 15 | 3 | 7 | 4 | 20 | 9 | 5 | 2 | 50 |
| 16 | 4 | 3 | 3 | 4 | 1 | 1 | 0 | 16 |
| 17 | 2 | 9 | 4 | 15 | 6 | 0 | 1 | 37 |
| 18 | 4 | 7 | 4 | 7 | 4 | 2 | 3 | 31 |
| 19 | 7 | 5 | 8 | 18 | 15 | 2 | 4 | 59 |
| 20 | 15 | 4 | 12 | 17 | 17 | 1 | 3 | 69 |
| 21 | 8 | 4 | 8 | 7 | 10 | 3 | 6 | 46 |
| 22 | 11 | 11 | 4 | 8 | 2 | 0 | 8 | 44 |
| 23 | 15 | 0 | 7 | 10 | 11 | 5 | 3 | 51 |
| 24 | 13 | 8 | 10 | 23 | 18 | 0 | 1 | 73 |
| 25 | 5 | 4 | 12 | 12 | 2 | 3 | 3 | 41 |
| 26 | 15 | 10 | 8 | 28 | 31 | 4 | 5 | 101 |
| 27 | 12 | 9 | 8 | 15 | 2 | 4 | 4 | 54 |
| 28 | 30 | 13 | 7 | 37 | 17 | 4 | 7 | 115 |
| 29 | 11 | 6 | 9 | 19 | 16 | 10 | 2 | 73 |
| 30 | 5 | 1 | 7 | 16 | 10 | 4 | 7 | 50 |
| 31 | 9 | 2 | 3 | 15 | 6 | 5 | 1 | 41 |
| 32 | 3 | 5 | 7 | 8 | 7 | 2 | 2 | 34 |
| 33 | 12 | 4 | 5 | 19 | 8 | 4 | 3 | 55 |
| 34 | 15 | 3 | 3 | 12 | 22 | 1 | 0 | 56 |
| 35 | 25 | 0 | 8 | 30 | 10 | 5 | 6 | 84 |
| 36 | 9 | 3 | 0 | 13 | 13 | 2 | 6 | 46 |
| 37 | 9 | 2 | 5 | 14 | 3 | 1 | 3 | 37 |
| 38 | 14 | 7 | 7 | 15 | 9 | 3 | 8 | 63 |
| 39 | 0 | 0 | 8 | 6 | 9 | 1 | 2 | 26 |
| 40 | 20 | 11 | 8 | 25 | 7 | 6 | 2 | 79 |
| 41 | 13 | 7 | 1 | 10 | 3 | 3 | 15 | 52 |
| 42 | 17 | 13 | 10 | 29 | 11 | 6 | 1 | 87 |
| 43 | 31 | 4 | 10 | 18 | 14 | 7 | 3 | 87 |
| 44 | 16 | 7 | 4 | 27 | 13 | 6 | 8 | 81 |
| 45 | 9 | 11 | 5 | 23 | 7 | 1 | 5 | 61 |
| 46 | 9 | 4 | 4 | 7 | 0 | 0 | 3 | 27 |
| 47 | 4 | 3 | 2 | 5 | 6 | 2 | 3 | 25 |
| 48 | 0 | 2 | 3 | 10 | 1 | 6 | 4 | 26 |
| 49 | 9 | 3 | 0 | 12 | 5 | 3 | 0 | 32 |
| 50 | 7 | 11 | 4 | 17 | 8 | 5 | 4 | 56 |
| 51 | 14 | 4 | 7 | 5 | 14 | 5 | 5 | 54 |
| **∑** | 487 | 299 | 284 | 680 | 410 | 135 | 188 | **2483** |

Values are *n.* A: Area according to the Bordeaux atrial region classification. ∑: Sum.

| **Supplementary Table 3: Median overall driver activity.** | | | | | | | | |
| --- | --- | --- | --- | --- | --- | --- | --- | --- |
|  | **A1** | **A2** | **A3** | **A4** | **A5** | **A6** | **A7** | **Overall** |
| **Number of focal activities per area** | | | | | | | | |
| Median (IQR) | 3 (2-6) | 2 (2-5) | 0 | 4 (2-8) | 2 (2-4) | 2 (2-2) | 0 | 6 (1-11) |
| **Number of rotor activities per area** | | | | | | | | |
| Median (IQR) | 9 (4-14) | 5 (3-8) | 5 (4-8) | 13 (6-19) | 8 (4-13) | 3 (2-5) | 4 (2-5) | 46 (27-61) |

A1-7: Area according to the Bordeaux atrial region classification. IQR: inter quartile range.

| **Supplementary Table 4: Concomitant disease specifics.** | |
| --- | --- |
| MR+TR | 16 (31) |
| MS/MR+TR | 6 (12) |
| MR | 6 (12) |
| MR+CAD | 2 (4) |
| MR+TR+CAD | 4 (8) |
| MR+TR+CAD+St.p. MVr | 1 (2) |
| MR+TR+AR | 2 (4) |
| MR+TR+Scimitar syndrome | 1 (2) |
| MR+TR+St.p. MitraClip | 1 (2) |
| MR+TR+AR+AAA | 1 (2) |
| MR+AR | 1 (2) |
| MR+AR+AAA | 1 (2) |
| MR+AR+St.p. MVr | 1 (2) |
| MR+AS | 1 (2) |
| AR+AAA+CAD | 1 (2) |
| AS/AR+AAA+CAD | 1 (2) |
| AS+AAA | 1 (2) |
| AS+AAA+CAD+St.p. ASD | 1 (2) |
| TR+AR+ St.p. MVR | 1 (2) |
| TR | 1 (2) |
| TR+MS+AS due to severe PPM | 1 (2) |

Values are *n* (%).

AAA: aneurysm of the ascending aorta; AF: atrial fibrillation; ASD: atrial septal defect; AR: aortic valve regurgitation; AS: aortic valve stenosis; CAD: coronary artery disease; COPD: chronic obstructive pulmonary disease; PPM: patient prosthesis mismatch; MR: mitral valve regurgitation; MS: mitral valve stenosis; MVr: mitral valve repair; MVR: mitral valve replacement; NYHA: New York Heart Association; TR: tricuspid valve regurgitation.

**Supplementary Figure 1:** Mapping study of patient 21 showing the distribution of drivers in LAO and PA view.


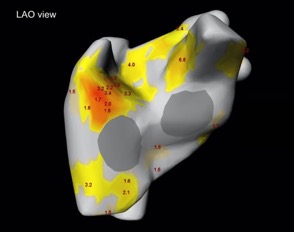

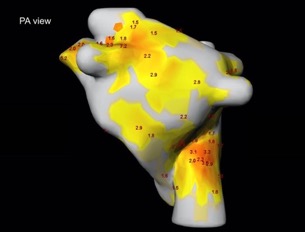


The focal activities are shown as orange diamonds while rotor activity is depicted as yellow/orange area. The number at the area’s center indicates the number of rotations. LAO: left anterior oblique; PA: posterior anterior.

**Supplementary Figure 2**: Relationship between AF duration and the number of rotor and focal activity.
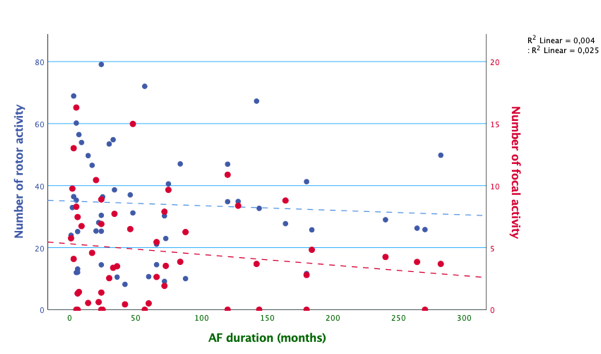


Every red and blue dot represents a patient. Red dots indicate the number focal and blue dots the rotor activity in relation to the patients AF duration in months.

**Supplementary Figure 3:** Dendrogram of the hierarchical cluster analysis.


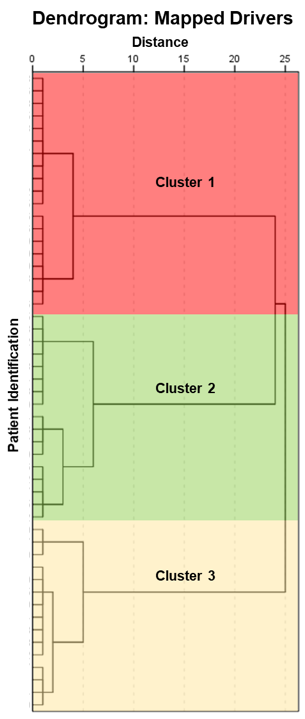


This dendrogram was used to establish the number of clusters for input into the k-means clustering algorithm. Eight iterations were used to minimize variances within clusters. After each iteration, the algorithm dynamically adjusted the centroid values, continuing until they converged and remained constant
